# Spatial mapping of tumor heterogeneity in whole-body PET–CT: a feasibility study

**DOI:** 10.1186/s12938-023-01173-0

**Published:** 2023-11-25

**Authors:** Hanna Jönsson, Håkan Ahlström, Joel Kullberg

**Affiliations:** 1 Section of Radiology, Department of Surgical Sciences, Uppsala University, 751 85 Uppsala, Sweden; 2 Antaros Medical AB, BioVenture Hub, 431 53 Mölndal, Sweden

**Keywords:** Tumor distribution, Tumor heterogeneity, Voxel-wise analysis, Whole-body PET–CT

## Abstract

**Background:**

Tumor heterogeneity is recognized as a predictor of treatment response and patient outcome. Quantification of tumor heterogeneity across all scales may therefore provide critical insight that ultimately improves cancer management.

**Methods:**

An image registration-based framework for the study of tumor heterogeneity in whole-body images was evaluated on a dataset of 490 FDG-PET–CT images of lung cancer, lymphoma, and melanoma patients. Voxel-, lesion- and subject-level features were extracted from the subjects’ segmented lesion masks and mapped to female and male template spaces for voxel-wise analysis. Resulting lesion feature maps of the three subsets of cancer patients were studied visually and quantitatively. Lesion volumes and lesion distances in subject spaces were compared with resulting properties in template space. The strength of the association between subject and template space for these properties was evaluated with Pearson’s correlation coefficient.

**Results:**

Spatial heterogeneity in terms of lesion frequency distribution in the body, metabolic activity, and lesion volume was seen between the three subsets of cancer patients. Lesion feature maps showed anatomical locations with low versus high mean feature value among lesions sampled in space and also highlighted sites with high variation between lesions in each cancer subset. Spatial properties of the lesion masks in subject space correlated strongly with the same properties measured in template space (lesion volume, *R* = 0.986, *p* < 0.001; total metabolic volume, *R* = 0.988, *p* < 0.001; maximum within-patient lesion distance, *R* = 0.997, *p* < 0.001). Lesion volume and total metabolic volume increased on average from subject to template space (lesion volume, 3.1 ± 52 ml; total metabolic volume, 53.9 ± 229 ml). Pair-wise lesion distance decreased on average by 0.1 ± 1.6 cm and maximum within-patient lesion distance increased on average by 0.5 ± 2.1 cm from subject to template space.

**Conclusions:**

Spatial tumor heterogeneity between subsets of interest in cancer cohorts can successfully be explored in whole-body PET–CT images within the proposed framework. Whole-body studies are, however, especially prone to suffer from regional variation in lesion frequency, and thus statistical power, due to the non-uniform distribution of lesions across a large field of view.

**Supplementary Information:**

The online version contains supplementary material available at 10.1186/s12938-023-01173-0.

## Background

Tumor heterogeneity is a term used to describe how the same type of cancer may manifest differently in different patients. Heterogeneity also exists on patient-level in the form of different subpopulations of tumor cells, each with distinct characteristics, within a single tumor lesion. There is both spatial heterogeneity, which describes the spatial organization of tumor cells, and temporal heterogeneity that describes the evolution of different cell populations over time as the disease progresses [1]. Tumor cells can only progress to other sites once they have acquired certain features identified as hallmarks of metastasis [2]. Spatial and temporal heterogeneity directly affects response to therapy [3, 4]. Detection, characterization, and quantification of tumor heterogeneity is therefore needed to improve therapeutic outcomes [5] and advance towards personalized cancer medicine [6, 7].

There is a vast set of methods or technologies for quantification of tumor heterogeneity across different scales [8]. Most techniques, however, relate to the study of molecular and architectural heterogeneity on cell or tumor level in tissue samples. Organ- and patient-level heterogeneity, as measured with imaging modalities, commonly, computed tomography (CT) and positron emission tomography (PET), is less studied. On this scale, heterogeneity manifests as variability in location, X-ray attenuation, tracer uptake, and shape descriptors (e.g., volume). Tumor volume is a strong predictor of radiotherapy outcome [9] and may also impact response to immunotherapy [10]. One advantage with imaging over tissue sampling is that every tumor lesion can be studied in both space and time non-invasively. A branch of medical imaging processing, called radiomics, has emerged as a tool to computationally characterize tumor lesions in terms of features that correlate with phenotype and patient outcome [11, 12]. Radiomics features may be extracted from within the tumor itself, the tumor margins, and also surrounding healthy tissue for use in predictive or prognostic models [13, 14]. Radiomics studies have, however, mainly been limited to local or loco-regional cancer, i.e., features are only computed from one lesion. In the context of metastatic cancer, it is common to examine a subset of isolated lesions, but this approach may not accurately represent the full extent of tumor heterogeneity within an individual patient [15]. Thus, radiomics is at best a powerful tool to describe prognostic features of individual tumor lesions, but does not describe the spatiotemporal relationship between all available lesions in whole-body images. The spatiotemporal location of disease correlates with both clinical and molecular characteristics [16–18]. Initial tumor site is also emerging as an important prognostic factor itself, for example, if it is right- or left-sided, in metastatic colorectal cancer and lung cancer [19, 20]. A detailed description of the spatiotemporal tumor distribution in cancer cohorts could thus become increasingly important for patient stratification. Since whole-body PET–CT or CT imaging is performed in clinical routine of a majority of cancer patients, such images present a great opportunity for the study of spatiotemporal tumor heterogeneity.

In this work, we apply for the first time a spatial mapping of lesions in whole-body PET–CT images of cancer patients for the study of tumor heterogeneity. Manually segmented lesion masks from patients with lung cancer, lymphoma, or melanoma were assigned with image-derived features of the disease and mapped to a template space using image registration. In template space, the spatial relationship between these features in the body was analyzed. The aim of the study was to investigate potential use-cases and limitations of the proposed framework and evaluate the applied image registration method within the context of lesion studies.

## Results

**Fig. 1:**
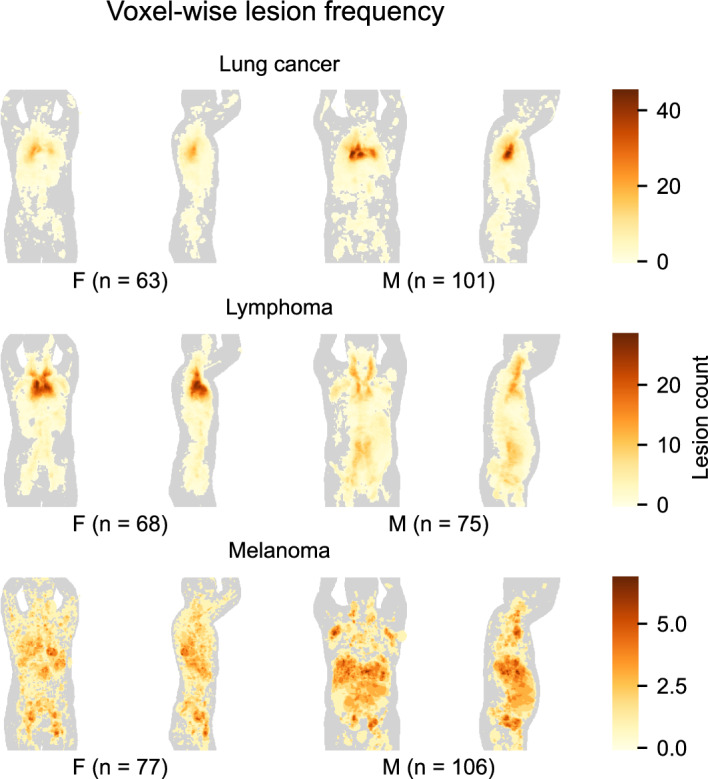
Voxel-wise lesion frequency maps of female (F) and male (M) patients. Lung cancer (top panel), lymphoma (mid panel), and melanoma (bottom panel) patients are presented separately. Subset sample sizes are printed below each panel. High lesion count is mapped to dark color. Images are coronal and sagittal maximum intensity projections

Figure 1 shows voxel-wise lesion frequency maps of the three cancer subsets separately. In the lung cancer subset (Fig. 1, top panel), lesion count is observed higher in the right lung than in the left lung (peak value *n* = 27 versus *n* = 18 in females; peak value *n* = 46 versus *n* = 32 in males). Maximum voxel-wise lesion count is lower in the female subset than in the male subset (*n* = 27 versus *n* = 46). In the lymphoma subset (Fig. 1, middle panel), the lesion frequency map of females shows a high concentration of lesions in the chest cavity. The lesion frequency map of males shows a more uniform distribution of lesion count in the body. Maximum voxel-wise lesion count is higher in the female subset than in the male subset (*n* = 29 versus *n* = 17). Peak value is observed in the chest cavity of females and in the neck region of males. In the melanoma subset (Fig. 1, bottom panel), both female and male lesion frequency maps have relatively low lesion counts per voxel compared to the other two subsets (peak value *n* = 6 in females; peak value *n* = 7 in males). Melanoma lesions are scattered throughout the body. A lower number of abdominal lesions in the female than in the male subset is seen. Above described relative differences between female and male subsets are also found in percentage frequency distribution maps (see Additional file 1).

**Fig. 2:**
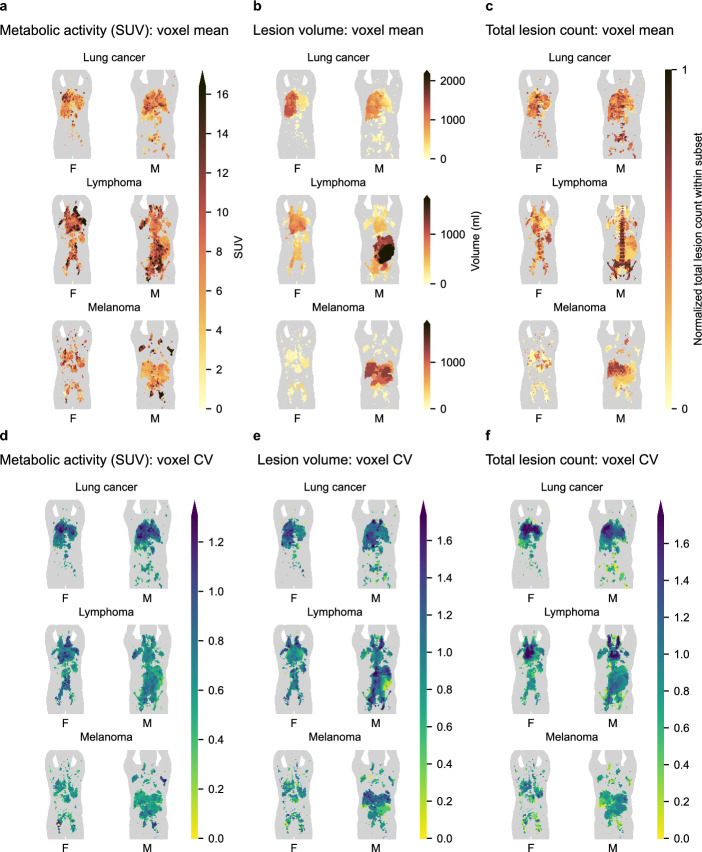
Voxel-wise lesion feature maps of features measured in subject space and summarized across female (F) and male (M) patients in template spaces. In each subplot, lung cancer (top panel), lymphoma (mid panel), and melanoma patients (bottom panel) are presented separately. In **a** and **d**, the feature shown is metabolic activity measured voxel-wise in subject space. In **b** and **e**, the feature shown is lesion volume mapped to each lesion in subject space. In **c** and **f**, the feature shown is total lesion count mapped to each lesion in subject space. In **a**–**c**, each pixel shows the mean feature value among lesions sampled at that location in space. High feature value is mapped to dark color. In **d**–**f**, each pixel shows the coefficient of variation (CV) among lesions sampled at that location in space. High variation is mapped to blue and low variation to yellow. Images are coronal maximum intensity projections. *SUV* standardized uptake value

Figure 2 shows lesion feature maps of the three selected features included for this study. Sagittal projection images corresponding to Fig. 2 are provided as additional material (see Additional file 2). Differences noted between the cancer subsets, and also between female and male subsets, are described in more detail below.

Figure 2a, d shows the results of voxel-level analysis of metabolic activity. In the lung cancer subset, FDG-uptake is relatively uniform in space (Fig. 2a, top panel). A smaller region with relatively high variation in uptake is seen in the right upper lung field in both females and males (Fig. 2d, top panel). In the lymphoma subset, the activity map of females shows a left–right asymmetry in the neck region (Fig. 2a, middle panel). In the melanoma subset, lesions in the abdominal region in the male subset have a lower metabolic activity than lesions sampled in other parts of the body (Fig. 2a, bottom panel). Mean metabolic activity across the body (calculated from voxel values visualized in Fig. 2a) is higher in the lymphoma subset (6.7 ± 3.4 in females; 6.0 ± 2.5 in males) than in the lung cancer (4.1 ± 2.0 in females; 4.1 ± 1.9 in males) and melanoma subsets (5.7 ± 2.8 in females; 4.7 ± 3.2 in males).

Figure 2b, e shows the results of lesion-level analysis of lesion volume. In the lung cancer subset, lesion volumes are larger in the right lung field than in the left lung field (Fig. 2b, top panel), but there is a high coefficient of variation in the same region (Fig. 2e, top panel). In the lymphoma subset, lesion volume appears largest in the chest cavity and neck region in the female subset, but largest in the abdominal region in the male subset (Fig. 2b, middle panel). High coefficient of variation is noted in the neck region of both females and males, and to some extent also in lymph nodes that lie in front of the vertebral column near the aorta (Fig. 2e, middle panel). In the melanoma subset, relatively small lesion volumes are observed at most sites, except in the abdominal region of the male subset (Fig. 2b, bottom panel). Table 1 summarizes the lesion volume data shown in Fig. 2b across the body. Mean lesion volume is largest in the lung cancer subset and smallest in the melanoma subset.

**Table 1:** Lesion volume data summary

Diagnosis	Patient sex	Total lesion count	Lesion volume (ml)
Lung cancer	Female	706	22.5 (3.1) ± 100
Lung cancer	Male	1118	24.9 (3.1) ± 87.1
Lymphoma	Female	1263	16.5 (0.8) ± 82.6
Lymphoma	Male	1942	11.3 (0.5) ± 76.5
Melanoma	Female	2150	4.7 (0.7) ± 22.8
Melanoma	Male	1090	10.6 (1.1) ± 72.1

Values are presented on the format mean (median) ± standard deviation

*SUV* standardized uptake value

Figure 2c, f shows the results of subject-level analysis of total lesion count. Figure 2c shows that lesions of the spine and hips in the male lymphoma subset (middle panel) and liver lesions in the male melanoma subset (bottom panel) belong to subjects with on average higher lesion count than lesions in other locations. Figure 2c also indicates that mean total lesion count is higher among female lymphoma patients with splenic involvement (middle panel) and, to a lesser extent, higher among lung cancer patients with lesions in the right lung field than in the left lung field (top panel).

**Fig. 3:**
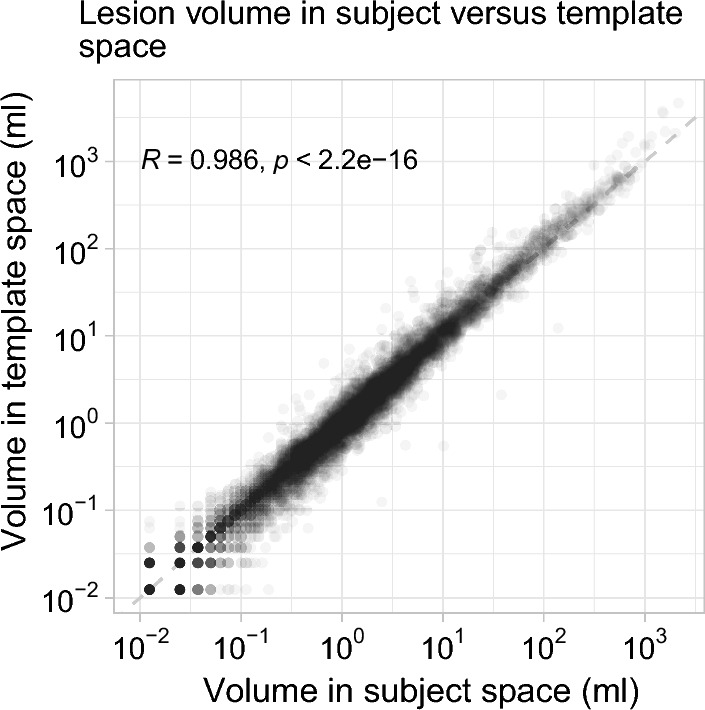
Lesion volume in subject versus template space. A scatter plot of lesion volume measured in subject versus template space for each lesion identified in the dataset. Each dot represents one lesion. Dashed line is an identity line. Axes are logarithmic

**Fig. 4:**
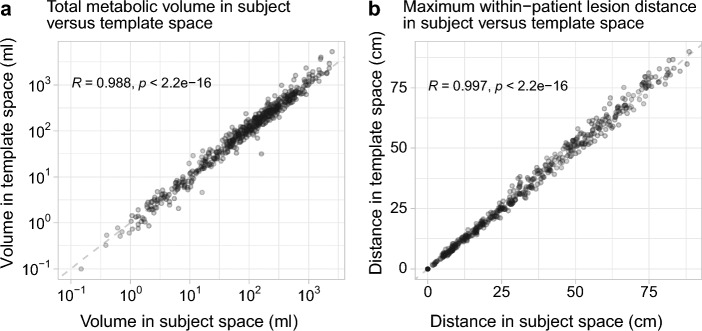
Spatial properties of the lesion masks in subject versus template space. Scatter plots of total metabolic volume (**a**) and maximum within-patient lesion distance (**b**) measured in subject versus template space for each subject in the dataset. Each dot represents one subject. Dashed line is an identity line. Axes are logarithmic in **a**

Figures 3 and 4 show the relationships between spatial properties of the subjects’ lesion masks before and after the mapping to template space. In Fig. 3, strong correlation between lesion volume in subject and template space is seen. Mean lesion volume change from subject to template space was 3.1 ml (median, 0 ml; standard deviation, 52 ml). There is also strong correlation between total metabolic volume in subject and template space (Fig. 4a). Mean total metabolic volume change from subject to template space was 53.9 ml (median, 5.2 ml; standard deviation, 229 ml). Mean pair-wise lesion distance change from subject to template space was − 0.1 cm (median, 0 cm; standard deviation, 1.6 cm) among all pairs of lesions (*n* = 794,018). Mean maximum within-patient lesion distance change from subject to template space was 0.5 cm (median, 0 cm; standard deviation, 2.1 cm). Figure 4b shows a strong linear relationship between maximum within-patient lesion distance in subject and template space.

## Discussion

In this study, we evaluated the feasibility of studying tumor heterogeneity in whole-body PET–CT images of cancer patients through an image registration-based framework. Spatial heterogeneity between the three studied subsets of cancer types, and also between female and male subsets, in terms of lesion frequency distribution in the body, metabolic activity, and lesion volume was shown. Additionally, a feature map of total lesion count could be used to visualize locations in the body more likely to be involved in advanced disease in the subsets. Lesion feature maps revealed differences between the subsets that could be confirmed when the data were summarized across the whole-body images. No systematic bias was found to be introduced by the mapping of lesions to template space.

Frequency maps of the three cancer subsets showed some differences between females and males. These differences could represent actual differences in female and male disease presentations, but there are also other possible explanations. One possibility is the presence of different subtypes of cancer in the dataset that have distinct frequency distributions. In lung cancer, differences, such as genetic mutations, have been reported in right- versus left-sided cancer [21, 22]. In lymphoma, involvement of the chest cavity is usually a sign of widespread disease, but most cases that begin there are a subtype known as Hodgkin’s disease [23]. In melanoma, the initial site of the lesion can influence its potential to spread to other parts of the body. For example, lesions in the head and neck region tend to preferentially spread to the liver [24]. Accordingly, an unequal sampling of such cancer subtypes between the female and male subsets would appear as different female and male lesion frequency distributions. Another interpretation of the sex differences seen is that there could be different proportions of early versus advanced stage disease in the female and male subsets. Unfortunately, subtype and stage descriptors of the studied dataset were unavailable, but this is of minor importance given the purpose of this study. Frequency maps presented here primarily illustrate the possibility to explore differences in lesion occurrences in the body between subsets of patients, and, most importantly, show that there is regional variation in lesion sample size on voxel-level. The latter finding needs to be taken into account in any future studies aimed at statistically comparing lesion distributions voxel-wise in whole-body images. Statistical power is a major concern also in voxel-based approaches to brain lesion-behavior correlations in functional neuroimaging [25].

Lesion feature maps exemplified in this study can provide different types of insight. Our study included both voxel-level and lesion-level features, as voxel-wise and regional analysis may provide important complementary information. Voxel-wise analysis is, in general, more easily affected by any systematic image registration errors, but can be especially useful for detecting focal differences [26, 27]. In the current study, the voxel-level feature map was indeed found to be most spatially detailed among the exemplified feature maps. Regional analysis will highlight patterns on a larger scale, which is illustrated clearly in Fig. 2 when the voxel-level feature map is compared with, most notably, the lesion-level feature map, but also the subject-level feature map. Subject-level analysis, here presented as a means to generate feature maps of global image descriptors of disease and their relation to tumor location in space, are tightly related to other lesion analysis methods. These techniques, often collectively called lesion-symptom mapping, have been applied mainly in neuroimaging, but also outside neuroimaging to limited fields of view. A similar approach was, for example, used to create survival status maps of locally advanced lung cancer [28]. In lesion-symptom mapping, patient groups are compared for voxel-wise statistical differences in lesion location in relation to some clinical variable of interest. Most commonly, a χ^2^-test for categorical variables and a t-test for continuous variables are used [29]. In the present study, the rationale for not comparing the patient group with lesion versus the patient group with no lesion voxel-wise is that our primary aim has been a description of the lesions sampled at each voxel location in space, i.e., a characterization of the lesion group only.

A strong relationship was found between subject and template space for the studied spatial properties of the subjects’ lesion masks. Our results suggest no general problem with the template selection and the applied image registration method, since average lesion volume change was less than 5 ml and average pair-wise lesion distance change was less than 1 cm from subject to template space. However, a relatively large difference between median and mean total metabolic volume change was noted. Outliers were also identified towards the far right in Fig. 3 that correspond to the largest lesions in the dataset. These lesions seem to more often have increased than decreased in size during the mapping to template space. They are, due to their volume, likely to exert a mass effect in the body and can thereby affect image registration results in the image region they occur in. This study implemented cost function masking during image registration which in case of a large tumor in, for example, the abdominal region, might make alignment of the whole abdomen of the subject with the template subject more difficult, since there was no penalty for mapping valid points in template space to the subject’s tumor region. Despite these known consequences of cost function masking, it is still the recommended approach in spatial mapping of brains with focal lesions, as unmasked registration may underestimate lesion volumes, especially of larger lesions [30]. Other approaches that have been found to improve image registration of lesioned brain images, include, for example, using cohort-specific templates [31] or using anatomical information from the side with no lesions [32]. These methods may be applicable to whole-body images to at least some extent and can be explored further in future studies. This could potentially improve registration accuracy for large lesions and possibly eliminate the need for their exclusion before subsequent analyses.

There are several advantages with the proposed framework to study tumor characteristics in whole-body PET–CT images. Most importantly, whole-body images can be fully utilized and studied, in contrast to with methods that only apply to a smaller field of view. This may improve the potential value of the large volumes of medical imaging data that are generated in healthcare each year [33, 34]. Its main feature is that it functions as a whole-body visualization tool that, as such, can intuitively describe the spatial location of lesions in the body and its relationship with features of interest. Features may be derived from images, as exemplified, and from multiple modalities, for example, both PET and CT features. In-depth analysis may involve exploring the spatial distribution of more sophisticated lesion features, such as first-order radiomics features. Studies are, however, not necessarily limited to image-derived metrics. For example, genomic data from known biopsy locations may be visualized as well. Temporal information may also be incorporated by first mapping a patient’s follow-up image to its baseline image to find out what has changed between the images and then map the resulting difference image to template space. Integration of different types data is identified as the key to building an accurate model of cancer and eventually being able to take better informed treatment decisions [35]*.* Image registration of PET–CT data from multiple cancer patients to a template space enables both visual and quantitative exploration of patterns that may only be visible after aggregating cohort data. A visual approach may also be more interpretable than other methods for feature extraction or fusion of biomedical image data and omics data [36]. Interpretability is especially important in healthcare, where a lack of such of is often seen to hinder clinical adoption of computational methods [8, 37]. Applications of the proposed framework can range from localized to metastatic cancer. Voxel-wise lesion studies of metastatic cancer are, however, as discussed above, more likely to suffer from low statistical power at any given patient sample size. In localized disease, whole-body image analysis may provide additional value in the form of a more comprehensive description of the patient’s disease status than image analysis applied to a limited field of view can. Even if the tumor is only localized, there might be other bodily changes related to the disease that are of study interest. Body composition, for example, visceral obesity and sarcopenia, is related to cancer outcome [38]. Computational analysis of texture features in images also has the potential to reveal signs of tissue damage before confirmed metastasized disease [39, 40]. Lastly, there are application domains of lesion frequency maps more oriented towards image processing. Tumor probability distributions may, for example, be used to improve automatic tumor segmentation [41].

This study’s main limitation is that the studied dataset lacked associated clinical variables other than main cancer type, patient sex, and age. Limited conclusions could therefore be drawn from the patterns discovered by the mapping of lesions in this particular dataset. Other properties of the dataset, such as, an adequate sample size of multiple cancer types, made it useful to illustrate a concept and therefore serve the purpose of our study. There are, however, also other limitations with our study related to the studied dataset. First, as the dataset analyzed was provided with lesion segmentation masks, our study could seem to diminish the pre-processing that must be applied to images before lesion mapping to a template space can be realized. If the proposed concept is to be applied to a previously unsegmented dataset, semi- or fully automatic segmentation methods can be used to reduce segmentation time compared with manual segmentation [42]. Secondly, the original dataset only included the segmentation of metabolically active lesions, and as a result, any non-metabolically active lesions in the CT scans could potentially affect the accuracy of image registration in regions where they may exist. Lastly, the evaluation of the mapping’s effect on lesion mask properties was for practical reasons limited to lesion volume and distances, i.e., one-dimensional measures. A more detailed technical evaluation of the applied image registration method, including other performance metrics, was previously performed on two other datasets with similar image acquisition parameters to the dataset in the current study [43].

## Conclusions

Our study suggests that the proposed framework can successfully be applied to explore spatial tumor heterogeneity in whole-body PET–CT images of cancer patients. Whole-body visualization and quantification of tumor heterogeneity, as illustrated, may be particularly useful for pattern extraction and hypotheses generation from cohort imaging data. It has the potential to be of great benefit for future studies aiming at understanding the association between tumor patterns in PET–CT images and patient outcome and ultimately the development of imaging biomarkers. Future studies should, however, expect a regional variation in lesion frequency, and thus statistical power, across the body that may also vary between subgroups of patients. Whole-body studies are especially prone to this due to the non-uniform distribution of lesions across a large field of view.

## Methods

### Dataset

A whole-body 18F-fluorodeoxyglucose-(FDG)-PET–CT dataset with manually segmented tumor lesions [44, 45] from the University Hospital Tübingen was analyzed in the study. The dataset is publicly available at The Cancer Imaging Archive [46] and comprised in total 1014 PET–CT examinations. PET images are provided with voxel values converted to standardized uptake value (SUV) based on body weight. There were 501 studies from patients with histologically proven lung cancer, lymphoma, or melanoma with at least one metabolically active tumor lesion (as defined by FDG-uptake on PET). These positive studies were included for the study of spatial tumor heterogeneity between the three subsets of cancer patients in the dataset. Remaining studies (*n* = 513) were from patients imaged with a clinical indication, but with no findings of metabolically active lesions. These negative controls were only used to select female and male template spaces to which all positive studies were mapped for voxel-wise analysis (see description below). From the original dataset of positive and negative studies, 22 subjects were excluded due to either one or two arms being positioned down during imaging (*n* = 21) or severe scoliosis (*n* = 1). These studies were excluded due to expected difficulties in achieving a satisfactory anatomical alignment with the template spaces. The final dataset comprised 490 positive studies and 502 negative controls. Of the positive studies, 164 studies were from patients with lung cancer, 143 studies were from patients with lymphoma, and 183 studies were from patients with melanoma. There were nine subjects with repeated imaging in the subset of positive studies; two studies (*n* = 7), three studies (*n* = 1), and four studies (*n* = 1). Patient characteristics of the final dataset are summarized in Table 2.

**Table 2:** Patient characteristics

Diagnosis	Patient sex	Number of studies	Age at imaging (years)
Lung cancer	Female	63	64 (62) ± 9 (48–83)
Lung cancer	Male	101	67 (67) ± 9 (44–83)
Lymphoma	Female	68	45 (44) ± 20 (15–79)
Lymphoma	Male	75	48 (50) ± 18 (11–85)
Melanoma	Female	77	65 (63) ± 13 (30–95)
Melanoma	Male	106	65 (65) ± 13 (19–89)
Negative	Female	228	59 (60) ± 15 (18–84)
Negative	Male	274	59 (61) ± 16 (18–85)

Values are presented on the format mean (median) ± standard deviation (min–max)

### Image registration

Image registration of the positive studies was performed using a registration method previously evaluated on two whole-body PET–CT datasets [43]. In the previous study, the assessment covered the anatomical matching and consistency of this registration method in both within- and between-subject registration tasks. The current study is a follow-up that places a particular emphasis on the registration of images containing lesions. All images were resampled to a slice thickness of 3 mm using linear interpolation, before undergoing further pre-processing and registration following the referenced method. Cost-function masking around lesions, as described in [43], was applied to preserve the original shape of the lesions. Female and male subsets were registered separately to a template space defined by one image in each subset. Template spaces were chosen from the subset of negative control patients based on image body fat percentage. Image body fat percentage was calculated as the percentage of voxels assigned as adipose tissue (− 190 to − 30 Hounsfield units) inside the torso part of each subject’s image. The torso was used, instead of the whole image, since there were field of view differences in the dataset. Median image body fat percentage was 44.6% for the female subset and 36.8% for the male subset. Female and male template spaces were selected from subjects whose image body fat percentages deviated by at most 2 percentage points from the respective subset medians. These template space images were chosen to ensure they did not contain any abnormalities that could introduce bias during image registration.

### Lesion feature map generation

First, lesion frequency maps were generated for the three cancer subsets to determine how many occurrences of lesions there were at each voxel location in the body. Segmented lesion masks were transferred to template space using the transforms resulting from image registration described in the previous section. Nearest neighbor interpolation was used during resampling to generate binary lesion masks in template space. In template space, lesion frequency maps were generated by counting the number of occurrences of lesions voxel-wise in each subset. Percentage frequency distribution maps were also generated by normalizing the frequency maps with the number of subjects in each subset.

**Fig. 5:**
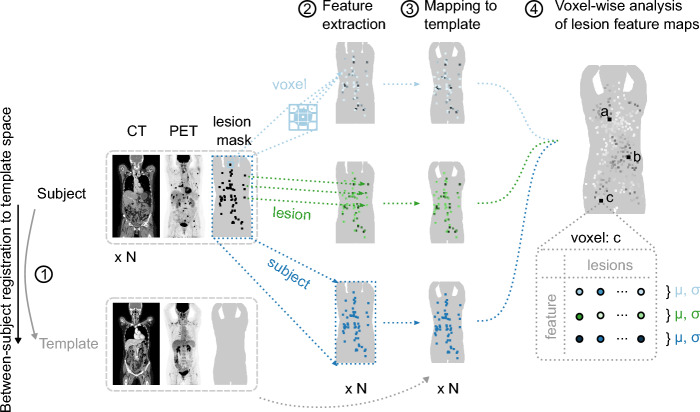
Illustration of method used to generate lesion feature maps in template space. Each subject's PET–CT image is first registered to template space (1). Voxel-, lesion- and subject-level features are extracted from the subject's lesion mask and used to construct lesion feature maps in subject space (2). Lesion feature maps are subsequently mapped to template space using image registration results (3). In template space, feature values are summarized across lesions sampled at each voxel location in the body (4)

Next, three types of feature maps were generated in template space to explore spatial tumor heterogeneity between and within the cancer subsets. Analysis was limited to voxel locations in template space with at least two occurrences of lesion in each female and male subset, respectively. Figure 5 illustrates the process. Each subject’s lesion mask was first labeled in subject space using a three-dimensional structuring element with connectivity 2, resulting in a lesion label map. Feature maps were then generated in subject space by assigning voxels of the lesion label map with the feature value of interest and then transferred to template space. Each lesion’s corresponding location in template space could be found as described above for the lesion frequency maps. In template space, voxel-wise mean and coefficient of variation were computed from the aligned feature maps of all subjects in each cancer subset. At each voxel, averaging and standard deviation calculation was performed only on lesions sampled at that location, i.e., subjects with no lesion at a particular voxel location would not contribute to the statistics. Resulting feature maps in template space thus describe the statistics among lesions at different positions in the body. The three types of feature maps studied include image intensity values on voxel-level (voxel-level analysis), features computed from each labeled lesion individually (lesion-level analysis), and features summarizing the segmented lesion mask on subject-level (subject-level analysis). Exemplified maps for the purpose of this study were voxel-level analysis of metabolic activity (in SUV), lesion-level analysis of lesion (metabolic) volume, and subject-level analysis of total lesion count. Total lesion count was scaled to 0–1 range by dividing the count by the maximum value in each female and male subset to improve comparability across the subsets. Successful image registration to the template spaces and subsequent lesion feature map generation should yield whole-body feature maps with enough spatial detail to discern similarities and differences among the three studied cancer types. The expected identifiable patterns include variations in the distribution of lesion frequencies throughout the body, the level of metabolic activity in these lesions, the size of the lesions, and the total count of lesions. In particular, the total lesion count is expected to correlate with disease progression and should enable visualization of body locations more likely to be involved in advanced disease in the subsets. Additionally, potential differences may be observed between the female and male subsets.

### Evaluation of the effect of image registration on spatial properties of lesion masks

Applying image registration to map lesions from subject to template space might distort lesions in size or change the pair-wise distances between lesions. To investigate such effects, spatial properties of the lesion masks were measured in subject space and compared with the same properties in template space after the mapping. Spatial properties included for study were lesion volume, total metabolic volume, all pair-wise distances between lesions, and maximum within-patient lesion distance. Maximum within-patient lesion distance is a measure of lesion dissemination in space and was defined as the maximum value of all pair-wise distances between lesions in the body. The distance between any two lesions was defined as the distance between their centers of masses. Only lesions in subject space mapped to within the field of view of the template spaces were included in the analysis. Pearson’s correlation coefficient was used to evaluate the relationships between subject and template space for the variables lesion volume, total metabolic volume, and maximum within-patient lesion distance. Successful image registration to the template spaces is evidenced by a strong correlation between subject and template space for all studied spatial properties. Summary statistics (mean, median, and standard deviation) were obtained for all studied variables. An appropriately chosen template space should result in a close to zero average change of these properties across all subjects, since there will be both patients smaller and larger than the template in the dataset that will be scaled accordingly, including lesions, during the mapping to template space.

### Supplementary Information

**Additional file 1**: Voxel-wise lesion percentage frequency maps of female (F) and male (M) patients. Lung cancer (top panel), lymphoma (mid panel), and melanoma (bottom panel) patients are presented separately. Subset sample sizes are printed below each panel. Lesion count is normalized by the number of patients in each female and male subset. High percentage lesion count is mapped to dark color. Images are coronal and sagittal maximum intensity projections.

**Additional file 2**: Voxel-wise lesion feature maps of features measured in subject space and summarized across female (F) and male (M) patients in template spaces. In each subplot, lung cancer (top panel), lymphoma (mid panel), and melanoma patients (bottom panel) are presented separately. In (a) and (d), the feature shown is metabolic activity measured voxel-wise in subject space. In (b) and (e), the feature shown is lesion volume mapped to each lesion in subject space. In (c) and (f), the feature shown is total lesion count mapped to each lesion in subject space. In (a), (b), and (c), each pixel shows the mean feature value among lesions sampled at that location in space. High feature value is mapped to dark color. In (d), (e), and (f), each pixel shows the coefficient of variation (CV) among lesions sampled at that location in space. High variation is mapped to blue and low variation to yellow. Images are sagittal maximum intensity projections. SUV = standardized uptake value.

## Data Availability

The dataset analyzed during the current study is available in The Cancer Imaging Archive repository, https://doi.org/10.7937/gkr0-xv29.

## Abbreviations

CT: Computed tomography
FDG: Fluorodeoxyglucose
PET: Positron emission tomography
SUV: Standardized uptake value
