# Supplementary figures and images for: Spatial mapping of tumor heterogeneity in whole-body PET–CT: a feasibility study
Source: Biomed Eng Online. 2023 Nov 25;22:110. doi: 10.1186/s12938-023-01173-0 (PMC10675915; doi:10.1186/s12938-023-01173-0)

# Voxel-wise lesion percentage frequency

## Lung cancer

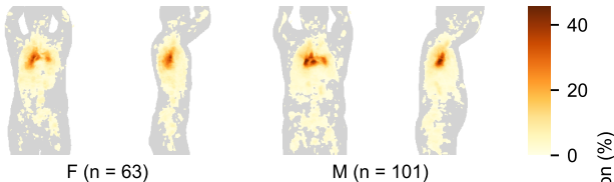

## Lymphoma

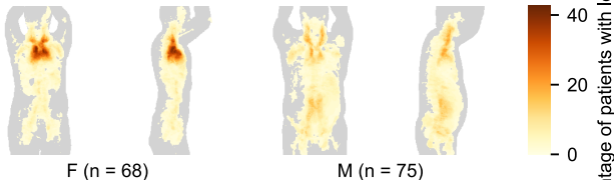

## Melanoma

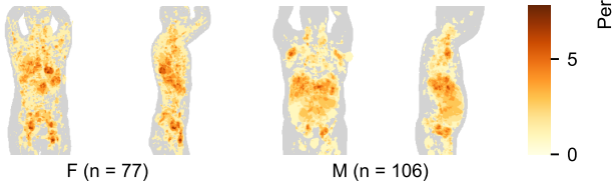

Supplement: Supplementary file 1 — Additional file 1: Voxel-wise lesion percentage frequency maps of female (F) and male (M) patients. Lung cancer (top panel), lymphoma (mid panel), and melanoma (bottom panel) patients are presented separately. Subset sample sizes are printed below each panel. Lesion count is normalized by the number of patients in each female and male subset. High percentage lesion count is mapped to dark color. Images are coronal and sagittal maximum intensity projections. [file 12938_2023_1173_MOESM1_ESM.pdf]

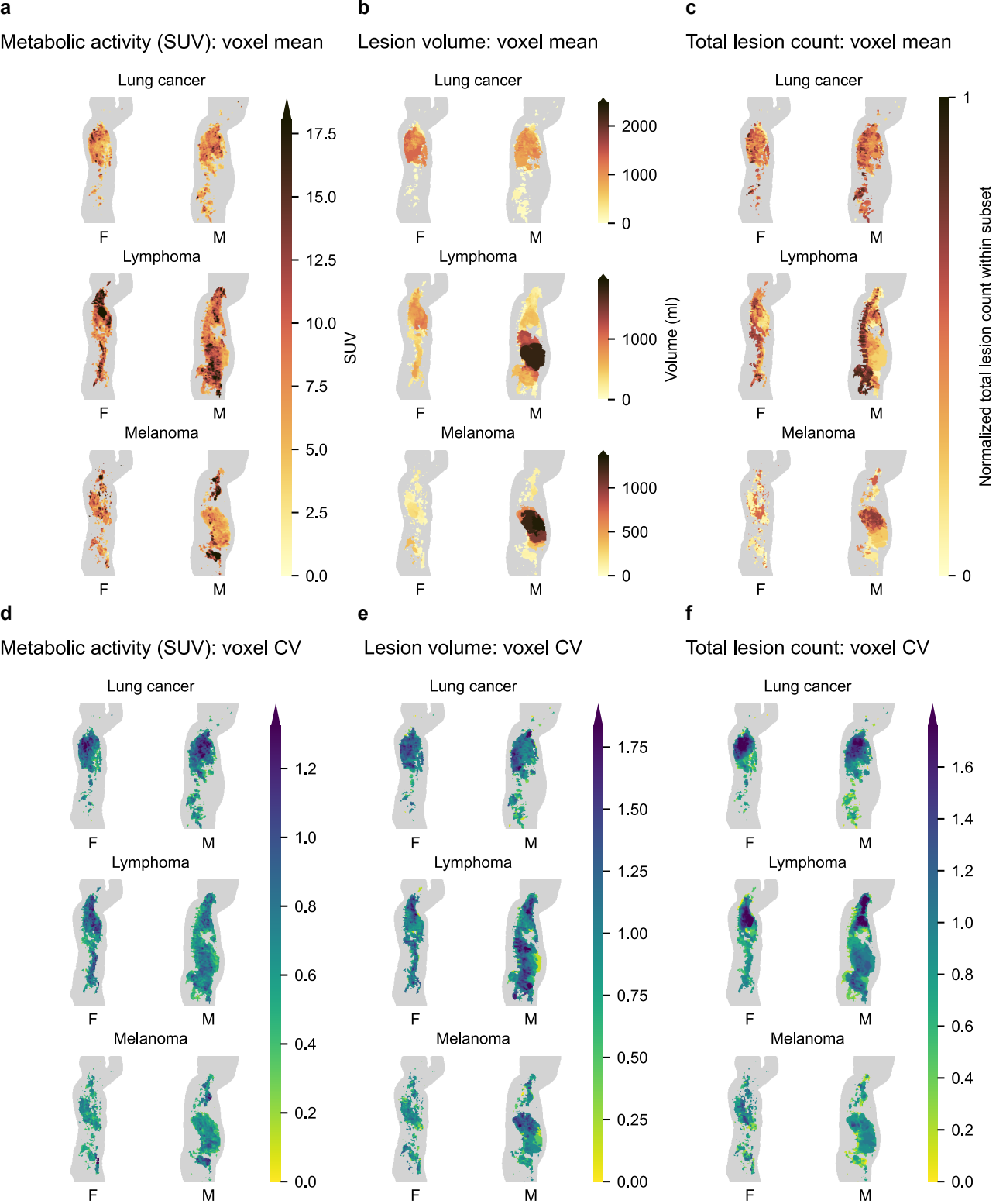

Supplement: Supplementary file 2 — Additional file 2: Voxel-wise lesion feature maps of features measured in subject space and summarized across female (F) and male (M) patients in template spaces. In each subplot, lung cancer (top panel), lymphoma (mid panel), and melanoma patients (bottom panel) are presented separately. In (a) and (d), the feature shown is metabolic activity measured voxel-wise in subject space. In (b) and (e), the feature shown is lesion volume mapped to each lesion in subject space. In (c) and (f), the feature shown is total lesion count mapped to each lesion in subject space. In (a), (b), and (c), each pixel shows the mean feature value among lesions sampled at that location in space. High feature value is mapped to dark color. In (d), (e), and (f), each pixel shows the coefficient of variation (CV) among lesions sampled at that location in space. High variation is mapped to blue and low variation to yellow. Images are sagittal maximum intensity projections. SUV = standardized uptake value. [file 12938_2023_1173_MOESM2_ESM.pdf]
